# Modulating the Structure of Graphitic Carbon Nitride for Accelerated Charge Separation and Enhanced Hydrogen Evolution

**DOI:** 10.3390/molecules31091458

**Published:** 2026-04-28

**Authors:** Kaijie Zhang, Yule Sun, Liuping Zheng, Guiyang Yan, Lu Chen

**Affiliations:** 1 College of Chemistry and Materials, Fujian Normal University, Fuzhou 350000, China; qsz20231468@student.fjnu.edu.cn; 2 Fujian Provincial Key Laboratory of Featured Materials in Biochemical Industry, Ningde Normal University, Ningde 352100, China; ygyfjnu@163.com; 3 State Key Laboratory of Photocatalysis on Energy and Environment, Fuzhou University, Fuzhou 350002, China; 241327088@fzu.edu.cn

**Keywords:** graphitic carbon nitride, barbituric, copolymerization, crystallinity, hydrogen evolution

## Abstract

Graphitic carbon nitride (CN) is considered a promising metal-free photocatalyst due to its adjustable electronic band structure and straightforward synthesis. Nevertheless, the practical utility of pristine CN is hindered by its rapid carrier recombination rate and low electrical conductivity. In this study, we enhanced CN’s molecular structure through copolymerization with organic molecules, thereby optimizing its crystallinity, resulting in significant improvements. The optimized photocatalyst, termed CNBM, demonstrated a remarkable hydrogen evolution rate of 23.13 mmol·h^−1^·g^−1^, a 118-fold increase compared to CN, with an apparent quantum efficiency of 87.9% at 420 nm. This notable enhancement in photocatalytic performance can be attributed to the increased surface area, providing more active sites, and the incorporation of barbituric acid through copolymerization into the CN framework, facilitating electron delocalization. Furthermore, the enhanced crystallinity of CNBM promotes the effective separation of photogenerated electron–hole pairs.

## 1. Introduction

Swift economic growth has led to excessive consumption of non-renewable fossil fuels and escalating environmental pollution, posing a significant challenge. Photocatalytic technology is increasingly recognized as a promising solution to both the energy crisis and environmental degradation. Hydrogen is widely acknowledged as a clean energy alternative to mitigate the aforementioned energy challenges. Thus, the imperative lies in the development of a highly efficient photocatalytic system for hydrogen production in the realm of photo-driven water splitting.

Graphitic carbon nitride (CN) has emerged as a highly promising material for efficient semiconductor photocatalysis, owing to its narrow band gap (2.7 eV), excellent thermal and chemical stability, and suitability for large-scale applications [1,2,3]. However, pristine CN faces challenges such as limited specific surface area, high carrier recombination rates, and inherently low conductivity, which significantly impede its practical utility. Various strategies have been explored to overcome these limitations, including morphological manipulation, elemental doping [4,5,6,7,8,9,10], heterojunction construction [11,12,13,14], introduction of carbon or nitrogen vacancies [15,16], dye sensitization, and copolymerization [17,18,19]. Among various strategies to overcome these limitations, copolymerization of barbituric acid with the CN precursor to form CNB has emerged as an effective method. This approach enables modulation of the electronic structure and enhances optical absorption within the visible light spectrum. Nevertheless, CNB polymers synthesized via thermal polymerization often exhibit low polymerization attributed to kinetic constraints, while frameworks with defects may serve as recombination centers, diminishing charge carrier transfer efficiency. To address these challenges, Wang et al. employed molten salts at high temperatures to facilitate copolymerization [20,21,22]. Subsequent efforts have successfully produced CCN polymers using the ionothermal method. The resulting CCN photocatalysts, characterized by reduced defects, exhibit enhanced charge carrier transfer efficiency, thereby bolstering their potential for accelerated photocatalytic applications.

In this study, barbituric acid and melamine were utilized as precursors for the synthesis of CNBM semiconductors employing LiCl/KCl salts. The resulting photocatalyst demonstrated significantly enhanced polymerization compared to pristine CN. Notably, the CNBM variant displayed a remarkable photocatalytic activity of 23.13 mmol^−1^ g·h^−1^, surpassing that of pristine CN by a factor of 118. Moreover, the CNBM photocatalyst achieved an apparent quantum efficiency (AQE) of 87.9% at 420 nm and demonstrated exceptional stability over four consecutive cycles, maintaining performance for the entire 12 h reaction. The superior performance of CNBM can be attributed to its substantial surface area, more negative conduction band (CB) position, and enhanced efficiency in the separation and transfer of photogenerated carriers. This study presents an environmentally friendly and straightforward approach for producing highly crystalline copolymerization photocatalysts, thereby contributing to advancements in the field of photocatalysis.

## 2. Results

**Figure 1:**
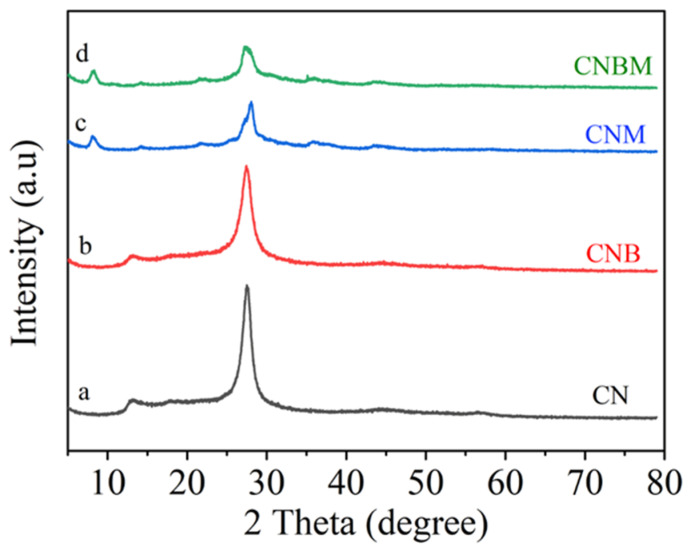
XRD patterns of (a) CN, (b) CNB, (c) CNM, (d) CNBM samples.

X-ray diffraction (XRD) analysis was employed to characterize the crystal structure of all photocatalysts. In Figure 1 (a), the diffraction peaks at positions 13° and 27° correspond to the (1 0 0) and (0 0 2) planes of pristine CN [23,24,25], respectively. The former plane was associated with interlayer packing features, while the latter plane was linked to interfacial stacking [26,27,28]. Following copolymerization with barbituric acid, the CNB sample exhibited a structure similar to that of CN (Figure 1 (b)). However, a decrease in peak intensity and broadening of peak shape were observed. These changes indicate a disruption of the orderly arrangement of CN due to copolymerization. Upon molten salt treatment (Figure 1 (c,d)), a notable shift in the (0 0 2) peak to 28° was observed, reflecting enhanced interlayer interactions and reduced interlayer distance [29,30,31]. Simultaneously, the (1 0 0) peak shifted to 8°, indicating an increase in in-plane periodicity [32]. The CNBM photocatalyst retained a layered structure, with significantly different in-plane periodicity compared to CN, confirming structural reconstruction.

**Figure 2:**
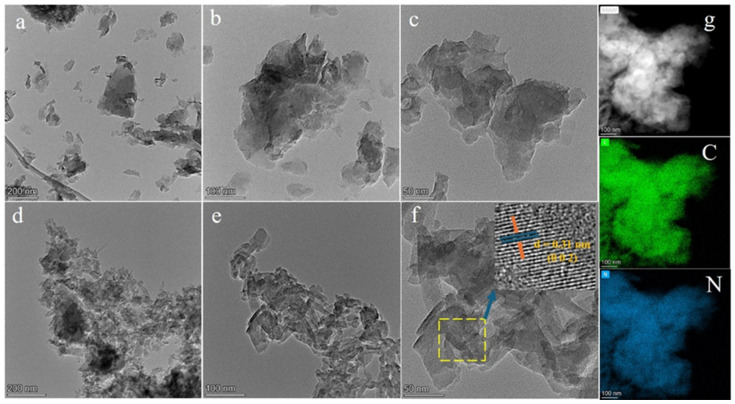
TEM characterization of (**a**–**c**) CN, (**d**,**e**) CNBM, HRTEM image of (**f**) CNBM, and (**g**) EDX mapping for C, N elements of the CNBM.

The morphology and microstructure of the photocatalysts were assessed using TEM and SEM techniques. As depicted in Figure 2a,b, the pristine CN exhibits a nanosheet structure with curled edges, comprising multiple layers of nanosheets. High-resolution TEM images indicate that the observed CN was in an amorphous phase (Figure 2c). Post-copolymerization, the CNB maintained a structure akin to that of CN (Figure S1). As illustrated in Figure 2d, the CNBM photocatalyst comprised small fragmented nanosheets, with distinct lattice fringes observed through HRTEM. The measured interplanar spacing of 0.31 nm for CNBM corresponded to the (0 0 2) plane [25], consistent with XRD findings. Additionally, EDX elemental mapping confirmed a uniform distribution of C and N within the material.

**Figure 3:**
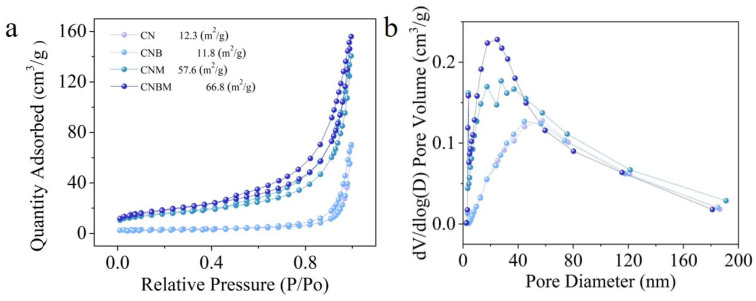
(**a**) N_2_ adsorption–desorption isotherms, (**b**) pore size distribution of four samples.

The specific surface area and pore volume were determined using nitrogen (N_2_) adsorption–desorption analysis. As shown in Figure 3, both samples exhibit characteristic type IV isotherms with H3 hysteresis loops, which indicate the presence of a mesoporous structure in the prepared photocatalysts. The CNBM sample exhibits a specific surface area of 66.8 m^2^·g^−1^ and a pore volume of 0.2 cm^3^·g^−1^, which are 5.4 and 2 times higher than those of CN (12.3 m^2^·g^−1^ and 0.1 cm^3^·g^−1^). The enhanced surface area and larger pore volume provide increased exposure to reactive sites for reactants, facilitating the diffusion of reactants and products and reducing mass transport limitations.

**Figure 4:**
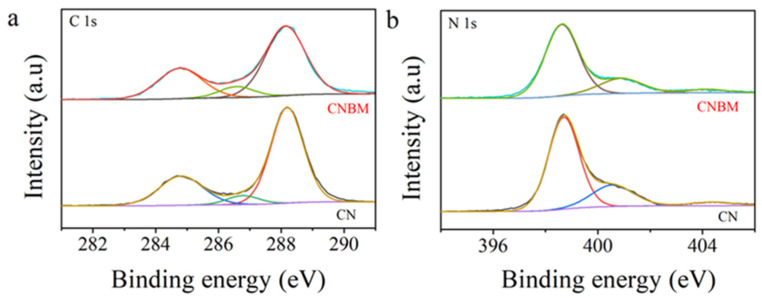
High-resolution XPS spectra of the as-prepared CN and CNBM samples: (**a**) C 1s and (**b**) N 1s.

X-ray photoelectron spectroscopy (XPS) was utilized to analyze the chemical states and elemental compositions of both CN and CNBM samples. As illustrated in Figure S2, the survey spectrum delineates the presence of C and N elements in both samples. In Figure 4a, the C 1s spectrum of CN exhibits three discernible peaks centered at 284.8, 286.7 and 288.2 eV. The peak at 284.8 eV is attributed to adventitious carbon species [33], while the peak at 286.7 eV is associated with C−NH species [34,35,36]. The peak at 288.15 eV corresponds to sp^2^ hybridized carbon (N−C−N) bonds [37,38]. In the N 1s spectrum (Figure 4b), the peaks at 398.7, 400.6, and 404.4 eV correspond to sp^2^-hybridized nitrogen (C−N=C group), tertiary N (C−N or C−N−H), and amino functional group (C–N–H) or the charging effect in heterocycles [39,40,41]. Notably, the binding energies of N 1s and C 1s in the CNBM sample were observed shift to lower positions, indicating a modification in the surface electronic structure due to molten salt treatment.

**Figure 5:**
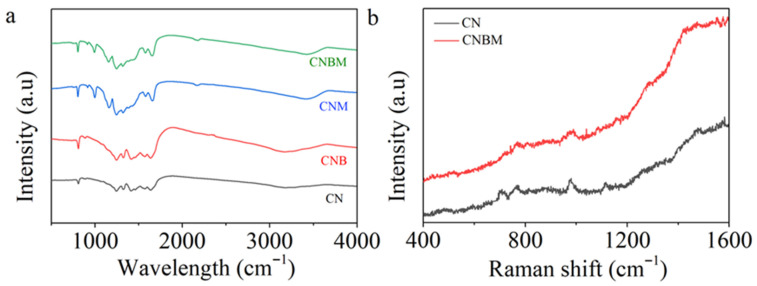
(**a**) FT-IR spectra of all samples, (**b**) Raman spectra of CN and CNBM.

The molecular structure of all prepared samples was further verified using FTIR analysis. In Figure 5a, the peaks ranging from 1000 to 1700 cm^−1^ are assigned to the C–N stretching vibrations in the CN heterocycle [42], with an additional peak at approximately 810 cm^−1^ attributed to the heptazine heterocyclic ring (C-N) [43]. The peaks in the 3000–3500 cm^−1^ range were identified as vibrational absorptions of N-H bonds and surface adsorbed hydroxyl (C-OH) groups [44,45,46]. Following copolymerization, the CNB sample maintained the original CN structure. However, both CNBM and CNM exhibited new peaks at 999, 1173, and 2170 cm^−1^ after molten salt treatment. The peaks at 999 and 1173 cm^−1^ were associated with the symmetric and asymmetric vibrations of NC bonds [36,47], while the peak at 2170 cm^−1^ corresponded to the cyano group (C≡N) [48,49,50]. In Figure 5b, the Raman peaks at 708 and 978 cm^−1^ are attributed to the in-plane bending vibration mode of the heptazine linkage and the symmetric N-breathing mode of the heptazine unit [51]. Additionally, the peak in the 1200–1600 cm^−1^ range was assigned to C-N stretching vibrations [52]. Overall, these results confirm that CNBM maintained a similar molecular structure to CN following copolymerization and molten salt treatment.

**Figure 6:**
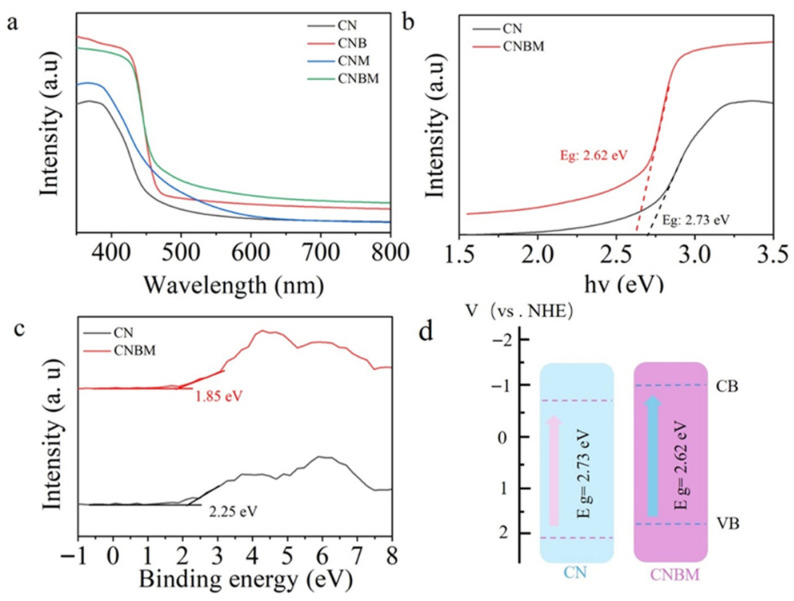
(**a**) UV-vis diffuse reflectance spectra of all samples, (**b**) the corresponding Tauc plots, (**c**) XPS-VB spectra, and (**d**) band structure of CN and CNBM.

The optical characteristics of the samples were analyzed using UV-vis absorption spectroscopy. The absorption spectrum in Figure 6a reveals a peak at 455 nm for CN. Upon copolymerization, the CNB sample exhibited increased absorption of visible light. Both CNM and CNBM samples displayed a blue shift in their absorption edges, indicative of the quantum confinement effect. The band gaps of the samples were determined through Kubelka–Munk analysis. In Figure 6b, the band gap energies are calculated as 2.73 eV for CN and 2.62 eV for CNBM. Evaluation of the valence band X-ray photoelectron spectroscopy (VB-XPS) plots for CN and CNBM estimated their respective VB potentials as 2.25 eV and 1.85 eV. Consequently, the VB values for CN and CNBM were determined as 2.05 eV and 1.65 eV, respectively, using the provided equation:

E_VB-NHE_ = ψ + E_VB-XPS_ − 4.44 (1)

The conduction band (CB) positions of CN and CNBM were determined to be −0.68 and −0.98 eV, respectively, based on the following equation:

E_CB_ = E_vb_ − Eg (2)

The CNBM photocatalyst demonstrates a lower conduction band (CB) potential compared to pristine CN, indicating its enhanced reductive capacity.

**Figure 7:**
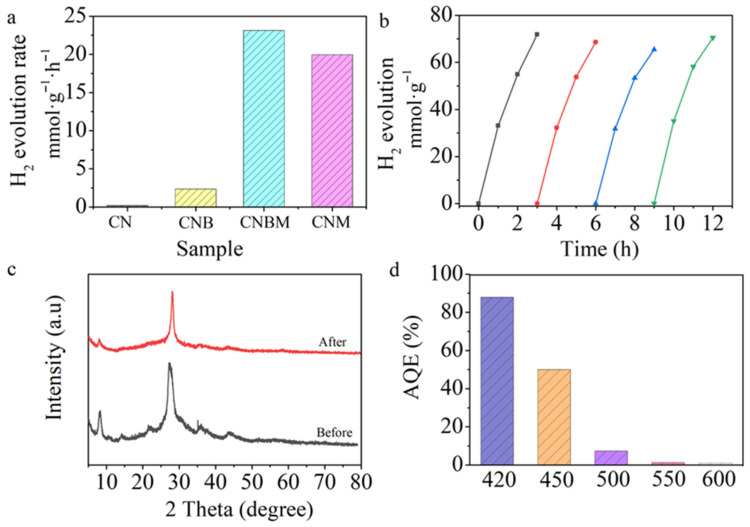
Photocatalytic hydrogen evolution rate (**a**) CN (S1), CNB (S2), CNBM (S3), CNM (S4), (**b**) cyclic hydrogen evolution test, (**c**) XRD patterns of sample before and after test, (**d**) AQE of CNBM.

The photocatalytic performance of all samples was evaluated in an aqueous solution with TEOA as a sacrificial agent. Figure 7a illustrates that the CNBM sample displayed superior photocatalytic activity of 23.13 mmol·g^−1^·h^−1^, surpassing CN, CNB, and CNM by factors of 118, 9.8, and 1.2, respectively. This enhancement underscores the significant improvement in photocatalytic efficiency following copolymerization and molten salt treatment. Notably, the photostability of a material is crucial for practical applications. Figure 7b demonstrates that the photocatalytic efficacy of CNBM remained constant over 12 h and four cycles. Additionally, X-ray diffraction (XRD) analysis in Figure 7c revealed no significant changes in the crystalline structure of the CNBM sample pre- and post-reaction, indicating its stability during the catalytic process. The apparent quantum efficiency (AQE) of CNBM was assessed at various wavelengths under identical conditions (Figure 7d), with AQE values at 420, 450, 500, 550, and 600 nm determined as 87.9, 50, 7.3, 1.2, and 0.9%, respectively. These results further confirm that the reactions were driven by photo absorption of the photocatalyst.

**Figure 8:**
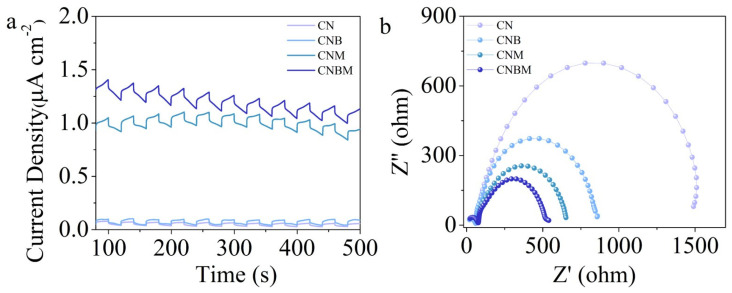
Photoelectrochemical test of all samples: (**a**) photocurrent response, (**b**) electrochemical impedance.

To understand the mechanism behind the excellent photocatalytic performance of CN and CNBM, analysis was conducted using electrochemical and photoluminescence (PL). As shown in Figure 8a,b, the CNBM sample displays a higher photocurrent density than pristine CN, revealing accelerated charge transfer and separation of photocarriers in CNBM. Meanwhile, the semicircle radius of the Nyquist plot of CNBM is remarkably smaller than that of CN, indicating the CNBM photocatalyst has a smaller electron transfer resistance. In addition, the PL peak intensity of CNBM was much lower than that of CN, revealing that CNBM has a lower recombination of photogenerated electron–hole pairs (Figure S3). Above all, it is further verified that the high separation and transfer efficiency of the CNBM sample are due to the high copolymerization with molecules (barbituric acid).

## 3. Materials and Methods

### 3.1. Materials

The chemicals utilized in this study include melamine, H_2_PtCl_6_·6H_2_O (with a Pt purity of 37.5% by weight), potassium chloride, barbituric acid, and lithium chloride monohydrate. All chemical reagents were used as received without further purification.

### 3.2. Preparation of CN

A specified amount of melamine was loaded into an alumina crucible equipped with a lid. Subsequently, the powder was heated at 550 °C for 4 h with a heating rate of 5 °C/min in a muffle furnace under an argon atmosphere. The resulting product was labeled as g-C_3_N_4_ (CN).

### 3.3. Synthesis of CNB

In a typical procedure, 10 g of melamine and 0.1 g of barbituric acid were dissolved in 30 mL of deionized water (DW) and stirred for 30 min. The resulting solution was then dried at 70 °C in an oven, followed by calcination in an alumina crucible at 550 °C for 4 h under a N_2_ atmosphere to yield the powder denoted as CNB. For comparison, CN was prepared using the same procedure, omitting barbituric acid.

### 3.4. Synthesis of CNBM

Then, 0.6 g of the CNB sample was thoroughly ground with 2.7 g of LiCl and 3.3 g of KCl. The resulting mixture was then calcined at 550 °C for 4 h, with a heating ramp of 5 °C/min, under a nitrogen atmosphere (60 mL·min^−1^). After cooling to room temperature, the obtained powder was washed several times with boiling water and subsequently dried overnight at 60 °C under vacuum. The resulting material was designated as CNBM.

### 3.5. Characterizations

The X-ray diffraction (XRD) analysis of the as-prepared photocatalysts was performed on a Bruker D8 instrument (Bruker AXS GmbH, Karlsruhe, Germany) equipped with a Cu Kα X-ray source to characterize their crystalline structures. The morphology and microstructure of the samples were assessed through transmission electron microscopy (TEM, JEOL Ltd., Tokyo, Japan) and scanning electron microscopy (SEM, Carl Zeiss AG, Oberkochen, Germany). The chemical compositions and states of the photocatalysts were determined using a VG ESCALAB 250 spectrometer (Thermo Fisher Scientific, Waltham, MA, USA) equipped with a monochromatized Al Kα X-ray source, with all binding energies calibrated to the C 1s peak at 284.8 eV. The optical properties were evaluated via UV–visible spectroscopy (Cary 500 spectrometer, Agilent Technologies, Santa Clara, CA, USA) with BaSO_4_ as a reflectance standard. Photoluminescence spectroscopy (PL) and time-resolved steady-state fluorescence lifetime measurements were performed using a fluorescence spectrophotometer (FSP920C, Edinburgh Instruments Ltd., Livingston, UK) with an excitation wavelength of 400 nm. Functional groups were identified using Fourier-transform infrared (FTIR) spectroscopy (Nicolet 670, Thermo Fisher Scientific, Waltham, MA, USA), while Raman spectra were acquired using an Invia Reflex Raman Microscopy system (Renishaw plc, Wotton-under-Edge, UK) with 325 nm laser excitation. The nitrogen adsorption–desorption isotherms, specific surface area, and pore size distribution were determined using an ASAP 2020 instrument (Micromeritics Instrument Corporation, Norcross, GA, USA).

### 3.6. Photoelectrochemical Measurements

The photoelectrochemical properties of all samples were assessed using an electrochemical station (CHI 6600, Shanghai, China) with a standard three-electrode cell configuration. Ag/AgCl electrode served as the reference electrode, while a Pt wire as the counter electrode. A layer of photocatalyst slurry was coated onto the conductive ITO glass surface and subsequently air-dried. Photocurrent measurements were performed in a 0.5 M Na_2_SO_4_ electrolyte under an applied bias of 0.5 V. Electrochemical impedance spectroscopy (EIS) was conducted over a frequency range of 0.01–10^5^ Hz using a 10 mM K_4_[Fe(CN)_6_]/K_3_[Fe(CN)_6_] solution.

### 3.7. Photocatalytic Test

The photocatalytic experiment was conducted using a vacuum-closed gas circulation system (Labsolar 6A, Perfectlight, Beijing, China). In a standard procedure, 50 mg of photocatalyst was dispersed in 100 mL of deionized water with 10% TEOA as a sacrificial agent and 0.25 g NaCl. Pt (3 wt%) was deposited on the photocatalyst surface as a co-catalyst via an in situ photo-deposition method using H_2_PtCl6·6H_2_O as a precursor. Following irradiation, the reaction system was vacuum-pumped for 10 min and stirred with a magnetic stirrer. The amount of hydrogen gas was analyzed using online gas chromatography (Techcomp, 7900, Ar carrier TCD detector, Shanghai, China). The apparent quantum efficiency (AQE) was determined under identical conditions and calculated using the following equation:

$$\mathrm{AQY}\;(\%)\;=\;\frac{\mathrm{Number}\;\mathrm{of}\;\mathrm{reacted}\;\mathrm{electrons}}{\mathrm{Number}\;\mathrm{of}\;\mathrm{incident}\;\mathrm{photons}}\;\times \;100\%\;=\frac{Number\;of\;evolved\;H_{2}\;molecules\times 2}{Number\;of\;incident\;photons}$$

## 4. Conclusions

In this study, we have successfully synthesized a highly crystalline metal-free carbon nitride-based material (CNBM) through copolymerization barbituric acid and subsequent molten salt treatment. The CNBM sample demonstrated a remarkable hydrogen evolution rate of 23.13 mmol·g^−1^·h^−1^ and an apparent quantum efficiency (AQE) of 87.9% under 420 nm light irradiation. Moreover, the CNBM sample exhibited exceptional stability over four consecutive cycles, maintaining its performance even after 12 h. This superior photocatalytic activity can be attributed to its large surface area, which provides abundant reactive sites, a negative conduction band potential favorable for hydrogen evolution, and high crystallinity that facilitates efficient separation and transfer of photogenerated electron–hole pairs. This study introduces a novel approach for producing high-crystalline, metal-free CNBM through copolymerization with barbituric acid molecules and molten salt treatment.

## Data Availability

Data are contained within the article.

## Supplementary Materials

The following supporting information can be downloaded at: https://www.mdpi.com/article/10.3390/molecules31091458/s1, Figure S1: SEM image of CNB, Figure S2: High-resolution XPS spectra of the as-prepared CN and CNBM samples: Survey, Figure S3: PL spectra of CN and CNBM.
