# Supplementary material for: Modulating the Structure of Graphitic Carbon Nitride for Accelerated Charge Separation and Enhanced Hydrogen Evolution
Source: Molecules. 2026 Apr 28;31(9):1458. doi: 10.3390/molecules31091458 (PMC13164741; doi:10.3390/molecules31091458)
Supplement: Supplementary file 1 [file molecules-31-01458-s001.zip › molecules-4240846-supplementary.pdf]

molecules

MDPI

Modulating the structure of graphitic carbon nitride for accelerated charge separation and enhanced hydrogen evolution

Kaijie Zhang[a,b], Yule Sun[c], Liuping Zheng[a*], Guiyang Yan[b], Lu Chen[b*]

[a] College of Chemistry and Materials, Fujian Normal University, Fuzhou 350000, P. R. China, qsz20231468@student.fjnu.edu.cn; zlpfjnu@163.com

[b] Fujian Provincial Key Laboratory of Featured Materials in Biochemical Industry, Ningde Normal University, Ningde 352100, P. R. China; T2110@ndnu.edu.cn; ygyfjnu@163.com

[c] State Key Laboratory of Photocatalysis on Energy and Environment, Fuzhou University, Fuzhou 350000, P. R. China; sunyule233@163.com

*Correspondence: T2110@ndnu.edu.cn;

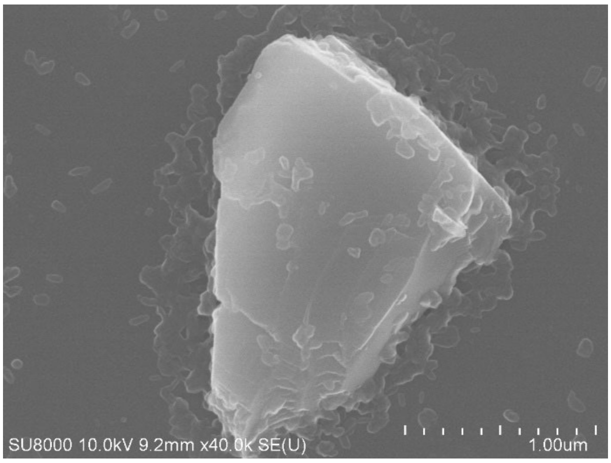

Figure S1. SEM image of CNB

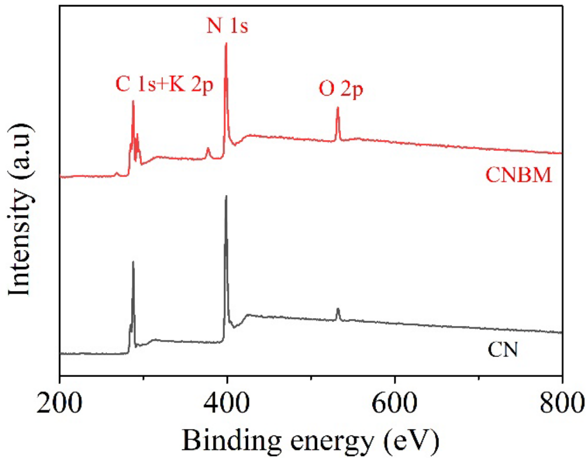

Figure S2. High-resolution XPS spectra of the as-prepared CN and CNBM samples: Survey

 https://doi.org/10.3390/10.3390/molecules31091458

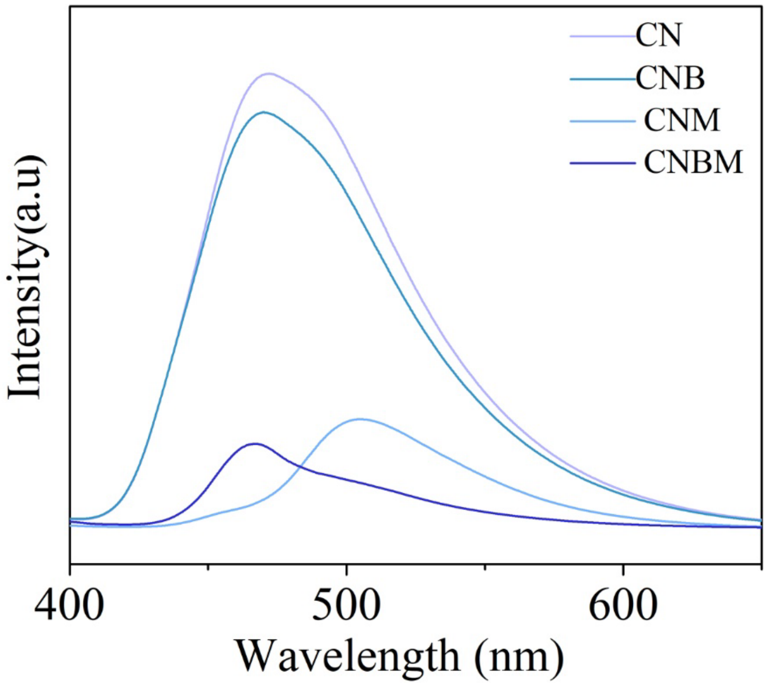

Figure S3. PL spectra of CN, CNB, CNM and CNBM
